# Multi-material Additive Manufacturing of Metamaterials with Giant, Tailorable Negative Poisson**’**s Ratios

**DOI:** 10.1038/s41598-018-26980-7

**Published:** 2018-06-14

**Authors:** Da Chen, Xiaoyu Zheng

**Affiliations:** Department of Mechanical Engineering, Virginia Tech, 635 Prices Fork Road, Blacksburg, VA 24061 USA

## Abstract

Nature has evolved with a recurring strategy to achieve unusual mechanical properties through coupling variable elastic moduli from a few GPa to below KPa within a single tissue. The ability to produce multi-material, three-dimensional (3D) micro-architectures with high fidelity incorporating dissimilar components has been a major challenge in man-made materials. Here we show multi-modulus metamaterials whose architectural element is comprised of encoded elasticity ranging from rigid to soft. We found that, in contrast to ordinary architected materials whose negative Poisson’s ratio is dictated by their geometry, these type of metamaterials are capable of displaying Poisson’s ratios from extreme negative to zero, independent of their 3D micro-architecture. The resulting low density metamaterials is capable of achieving functionally graded, distributed strain amplification capabilities within the metamaterial with uniform micro-architectures. Simultaneous tuning of Poisson’s ratio and moduli within the 3D multi-materials could open up a broad array of material by design applications ranging from flexible armor, artificial muscles, to actuators and bio-mimetic materials.

## Introduction

Materials with designed three-dimensional micro-architectures offer multiple beneficial properties such as low weight^1,2^, high stiffness and strength^1,3^, negative poisson ratio^4–7^ and energy absorptions^8–10^ and can open up a myriad of material by design applications from flexible armor^11,12^, responsive materials^13,14^ to bio-mimetic materials^15–18^. Ultimately, one would like to 3D print functional device or components that incorporate multiple material constituents without the requirement of excessive assembling procedures such as gluing, aligning, fitting, and welding. Apart from enhancing spatial resolution and printing speed, achieving this goal requires the ability to incorporate an array of different material properties within a manufacturing platform. In analogy to typical 2D color printers that can integrate multiple colors from mixing a few colors (magenta, cyan, yellow), a three-dimensional fabrication platform should not only be able to integrate multiple colors, but also be capable of spatially integrating encoded material properties and compositions from mixing only a limited number of feedstock materials.

**Figure 1:**
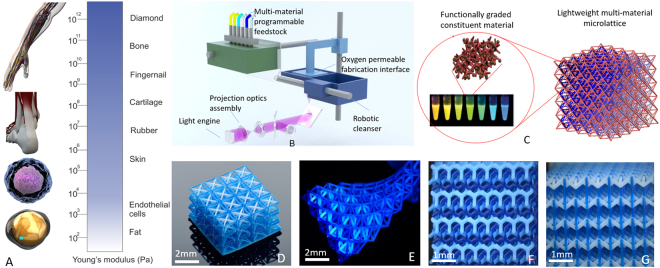
Fabrication of 3D multi-material microlattice with dissimilar constituent material (**A**) Schematic of 3D multi-material microlattice with encoded stiffness, (**B**) Experimental setup of modular digital light projection microstereolithgoraphy technique coupled with *in situ*-microfluidic systems for resin delivery. (**C**) As-fabricated bi-material lattice comprised of clear and yellow rigid polymer constituents. (**D**) As-fabricated isotropic re-entrant microlattice comprised of rigid polymer resin (blue) and ceramic polymer composite. (**F**) As-fabricated isotropic re-entrant microlattice comprised of a rubbery polymer and rigid polymer. (**E**) As-fabricated two-phase 3D gryoid separator.

Nature has evolved with a recurring strategy to achieve unusual mechanical properties through coupling large gradient elastic moduli from a few GPa to below MPa^19^. Biological tissue connecting tendons to bone exhibits locally tuned elastic moduli that can vary by as much as two orders of magnitude to match the stiff surface of bone with the soft tendon^20,21^ (Fig. 1A). Combining dissimilar mechanical properties within a material have been envisioned for a range of applications ranging from enhanced ductility and fracture toughness^22^, negative to zero thermal expansion^7,23–25^ and biomimetic materials.

While new designs and theories have been proposed to realize new property space as a result of heterogeneous composites^1,9,26–28^, experiments are limited by the lack of fabrication processes capable of building these three-dimensional microarchitectures with coded material properties. Dissimilar material joining has been limited to assembly of bulk components. Efforts have been developed over the past decades to create dissimilar materials using indirect joining, aerosol jet, multi-vat stereolithography, direct ink writing of different colors, or extrusion based techniques [24–26]. These techniques have been used to deposit different colors, hydrogels and inks with inorganic particles^29–35^. However, despite the ability of printing materials with different colors, these techniques have the challenge of creating complex 3D features of which the printing time scale up with the complexity of 3D architectures. Additionally, few techniques have been able to realize a full three-dimensional distribution of material properties over a complex, 3D architectures.

Fabrication of architected metamaterials of single materials, from polymers, to metallic and ceramic have seen considerable growth in the past decades on creating arbitrary shaped 3D architectures^1,36^. The fabrication of these microlattices is enabled by a high-resolution projection microstereolithography (PμSL), additive micro manufacturing process capable of fabricating arbitrary three-dimensional micro-scale structures^37,38^. In contrast to other 3D rapid prototyping methods such as 3D printing and UV projection waveguide systems^39^, this type of fabrication technology is ideal for 3D lattices with high structural complexity, and with feature sizes ranging from microns to centimeters^1,40^. Fabrication of these 3D architected metamaterials have been demonstrated ranging from tens of nanometers to tens of centimeters with arbitrary hierarchical architectures with printing speed independent of architectural complexity. The possibility of combining multiple materials in a complex, arbitrary three-dimensional geometry is an even more powerful asset that adds on an extra dimension in the available design space. However, the use of multiple materials in additive manufacturing presents challenges with managing contamination between material systems, which leads to blended colors and poor segregations of printed features  with distinct properties.

Here, we demonstrate a new class of multi-material metamaterials capable of achieving giant tunable Poisson’s ratios with coded spatially varying stiffness. These metamaterials are realized by a robotic multi-material additive manufacturing technique. In contrast to architected materials comprised of the same base material, these type of 3D multi-material metamaterials is comprised of distributed rigidity ranging from soft elastomeric to rigid brittle constituents with moduli spanning over two decades within a 3D lattice framework. These functionally graded feedstock materials with prescribed moduli can be directly coded by mixing monomers and photopolymerization into arbitrary 3D position inside a lattice framework. Production of these lattice structures with programmable material constituents from soft elastomeric to hard brittle polymer to functionally graded ceramic composite is made possible by a new digital light additive manufacturing technique capable of *in-situ* material delivery, cleansing and exchange robotic system (Fig. 1B). Figure 1(C) shows the schematics of functionally graded microlattice materials comprised of disparate material constituents coded in 3D microlattice.

## Results

**Figure 2:**
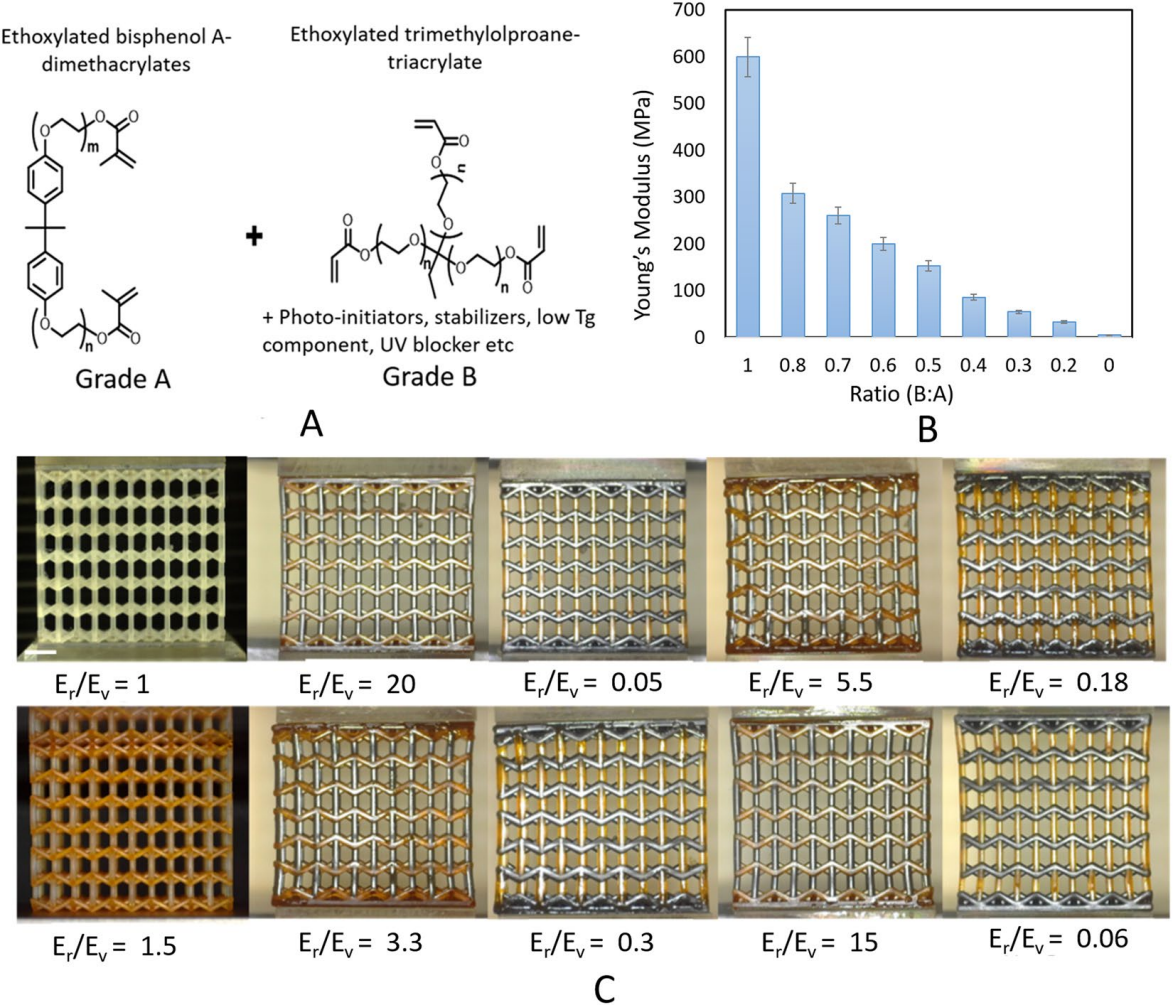
Constituent materials with large stiffness gradients. (**A**) Basic monomer composition that offer low and high stiffness. (**B**) Tunable bandwidth of stiffness in solidified photopolymers. (**C**) 3D printed multi-material microlattice with different stiffness ratio R varying between re-entrant and vertical strut members. Scale bar represents 2 mm.

Our additive manufacturing platform of heterogeneous feedstock is based on *in-situ* resin mixing and exchange and a robotic material cleansing system to achieve switching between materials with prescribed modulus without cross contamination between different properties (Fig. 1B). To demonstrate the printed properties, base materials with prescribed moduli have been encoded with different colors. To fabricate a multi-material three-dimensional object, each layer of an object is first divided into a number of parts (N) with corresponding moduli with a customized script (Supplementary Material S1). The process begins with deposition of the feedstock material onto an oxygen permeable membrane as the printing interface using a customized microfluidic delivery system. Ultraviolet patterns corresponding to patterns with property (i) is projected onto a monomer resin that can be loaded with functional nanoparticles or monomers with different cross-linking architectures (Fig. 2A). This induces a polymerization reaction that converts the liquid-state monomer resin of material (i) into a solid layer in the shape of the projected image (Supplementary information_S1). A new material is perfused using a self-cleansing robotic dispenser that allows simultaneous switching between different feedstock materials and cleaning residue monomer at each sequential layer before a new feedstock is perfused into the chamber. The process is repeated until the desired number of layers has been fabricated to complete the 3D object. In each layer the material switching time is 10 s including cleaning and drying time. The printing resolution, determined by the optics and the pixel size, is 5 μm, which is adjustable through the optics. A variety of microlattice architectures (re-entrant lattice, isotropic re-entrant lattice and 3D two-phase gyroid separator) are 3D printed with clear visualization of distinct materials (flexible, brittle, elastic polymers, and ceramic nanocomposite) as shown in Fig. 1D–G. Cleaning is completed by sweeping the residue materials with a brush (See Fig. 1). Different materials are printed within one layer. Our image sequence sequentially arrange the image pattern corresponding to different materials to be projected.

In our study, we modeled 3D architected re-entrant micro-architecture with variable moduli distribution within two orthogonal planes (e.g., E_r_ and E_v_) within the lattice. Examples of idealized unit cell is shown in Fig. 2B. Young’s modulus, yield strength, and Poisson’s ratio can be potentially varied within the lattice without changing the 3D geometric layout. Variable *θ* denotes the angle between two adjacent re-entrant struts and vertical strut; *H* denotes the height of vertical struts; *L* denotes the length of re-entrant struts and *t* denotes thickness (diameter) of the strut cross section. The ratio of the Young’s moduli between the two materials E_r_/E_v_ serves as the variable parameter controlling the bulk scale Young’s modulus and Poisson’s ratio of the microlattice material. This geometry-material design enables 3D architected materials with disparate modulus distribution.

The tunable feedstock materials are comprised of photo-curable monomers and oligomers that form tunable crosslinking architectures. As shown in Fig. 2, the large bandwidth of material properties is achieved by combining a very flexible oligomeric, polyether(meth)acrylate monomer with high molecular weight and a polyfunctional tri-acrylate with a lower molecular weight on the other end. Through varying the ratio between the triacrylate and diacrylate monomers, the Young’s moduli of the base materials can be tuned by assigning the hard, rigid material containing triacrylate momoner as the Grade A and methacrylate with a longer molelcular chain as the monomer as Grade B. The viscosity of the stock material used in this study have an estimated range 250–500 cps @ 25 °C. The tunable modulus of the photopolymer is prescribed through mixing between A and B as “E (i) = E_a_ A + E_b_ B” where A and B are the mixing ratios between the rigid and soft monomers (from a value of 0% to 100%), *i* is the location of the strut member within the metamaterial, respectively. This method makes it possible to produce base materials with a wide range of elastic properties in a simple process step while minimizing the polymerization depth (Fig. 2B). The elastic moduli of the constituent materials are all measured using ASTM standard for tensile (ASTM D638-10) and compression measurement (ASTM E111-04).

**Figure 3:**
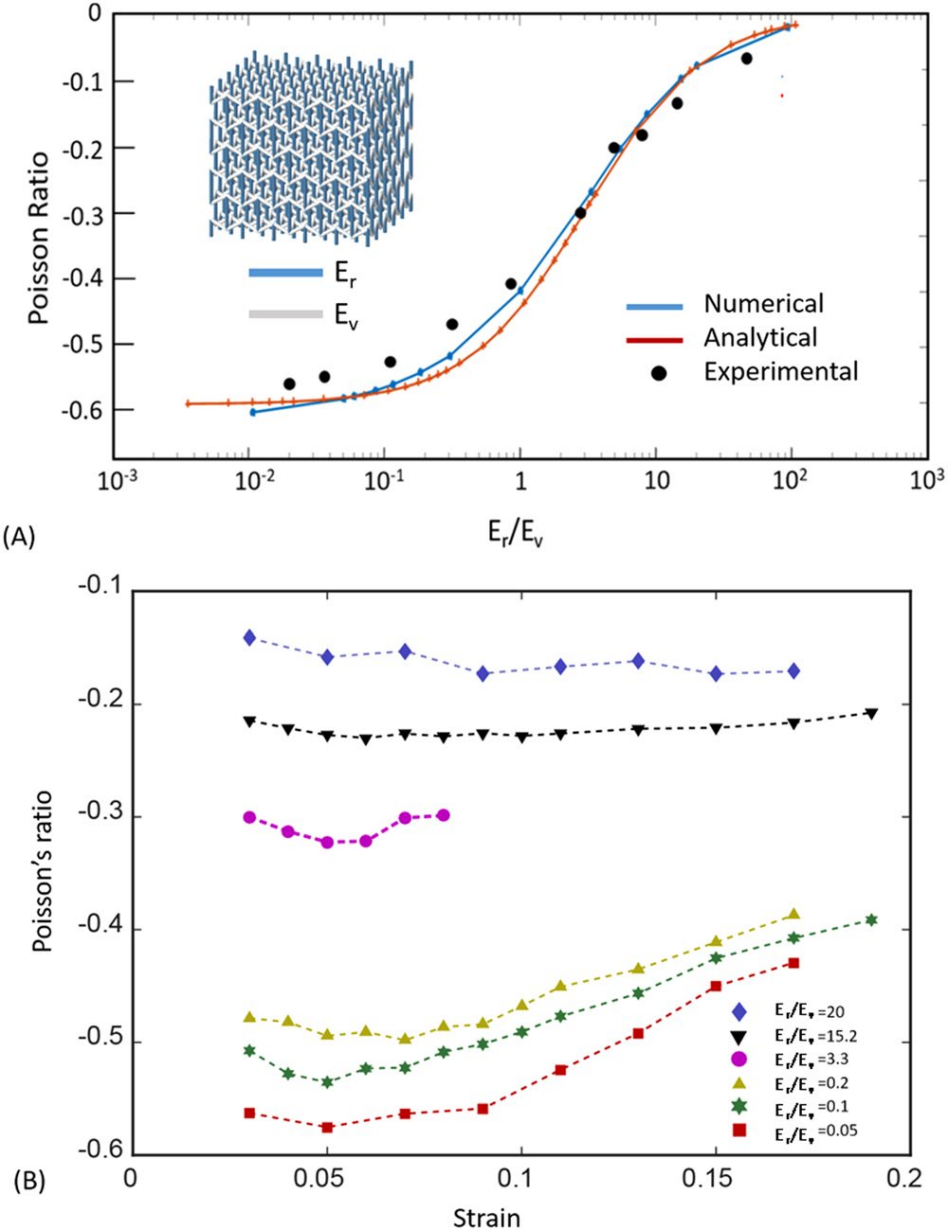
(**A**) Comparison between analytical, experimental and numerical calculation of tunable Poisson’s ratio from zero to negative as a function of dissimilar material stiffness ratio in an isotropic 3D re-entrant honeycomb metamaterial. (**B**) Effective Poisson ratios as a function of pulling strain in the longitudinal directions corresponding to metamaterial with different moduli ratios in 3D printed materials shown in Fig. 2C.

To demonstrate the variability of tunable Poisson’s ratio within the same 3D architecture, 3D microlattice with encoded Young’s modulus associated with different strut members within the 3D topology are fabricated. We assign E_r_ as the modulus of the re-entrant strut and modulus E_v_ as the vertical filament (Fig. 2B). Both E_r_ and E_v_ can vary within the established tunable modulus bandwidth. We denote the ratio between the re-entrant member to the vertical strut member as E_r_/E_v_, which can be tuned through the material grade between A and B constituent. A collection of as-fabricated 3D micro-architecture with varying E_r_/E_v_ values are shown in Fig. 2C, which are all comprised of identical 3D micro-architecture (*θ* = 60, H/L = 1.5) and same relative density. To investigate the mechanical deformation behaviors of the dissimilar materials, we fabricated specimen and conducted uniaxial tensile testing of the microlattices. As a point of comparison, a single material microlattice corresponding to the design parameter (*θ*, H/L, t/L) is fabricated and tested using the same method. The results of the uniaxial tensile tests with dissimilar microlattices are shown in Fig. 3A where measured the Poisson’s ratios are displayed as a function of E_r_/E_v_ ratio. Analytical and numerical investigation indicate that these 3D bi-material honeycomb microlattice can be tailored for desired effective Poisson’s ratios to suit different morphing application needs within a single homogenous 3D microarchitecture. Detailed analysis using Timoshenko bean theory and incorporation of different Young’s modulus in assigned bars is included in Supplementary materials. Here, the Poisson’s ratio for a 3D reentrant unit cell can be formulated by the following equations,

1 $${\upsilon }_{xz}=\,-\frac{{\rm{\Delta }}{x}_{r}(H-L\,\cos \,\theta )}{({\rm{\Delta }}{y}_{r}+{\rm{\Delta }}{y}_{v})L\,\sin \,\theta }$$

2 $${\upsilon }_{xz}=\,-\frac{(\frac{{L}^{2}}{{{\rm{4E}}}_{r}{t}^{4}}+\frac{3}{10{G}_{r}{t}^{2}})\sin \,\theta (\alpha -\,\cos \,\theta )}{\frac{\alpha }{{E}_{v}{t}^{2}}+(\frac{{L}^{2}}{{{\rm{4E}}}_{r}{t}^{4}}+\frac{3}{10{G}_{r}{t}^{2}}){(\sin \theta )}^{2}}$$

where, $=H/L$. ${E}_{v}$ and ${E}_{r}$ are the Young’s modulus of vertical and re-entrant strut respectively. ${G}_{r}$ is the shear modulus of re-entrant filament. All base material data were measured through tensile and compression testing on solid materials for each Er and Ev base material used in our study. The Poisson’s ratio is calculated by customized image analysis software written in MATLAB to calculate the deformations before and after deformations. It can be found from equation (2) that the Young’s modulus ratio ${E}_{r}/{E}_{v}$ plays an important role of describing NPR behavior of a re-entrant structure. We found that giant negative to zero Poisson’s ratio could be tuned to a regime not achievable through homogeneous material distribution (when ${E}_{r}/{E}_{v}=1$) (Fig. 3A). Finite element analysis was performed to verify our analytical model. A good agreement with analytical model can be seen in Fig. 3(A). For example, at a dissimilar ratio E_r_/E_v_ = 0.02 between two Young’s moduli, large negative Poisson effect (*v* ~ −0.6) can be clearly visualized, whereas at E_r_/E_v_ = 40, a close to zero poisson ratio effect is displayed with identical micro-architecture as in E_r_/E_v_ = 0.02. At a dissimilar ratio E_r_/E_v_ = 1 the microlattice corresponds to homogenous microlattice material displaying a Poisson ratio at a value of −0.3. Additionally, programmable Young’s modulus as well as shear modulus can be tuned through as the ratio between Er/Ev modulates from 0.01 to 100 (Fig S5).

Given the excellent qualitative and quantitative agreement found between our experiments and simulations, we further investigated the evolution of Poisson’s ratios as a function of pulling strain in z direction. Figure 3B plots the Poisson’s ratios in metamaterials with identical microarchitectures and varying modulus under large deformation. The variation under larger strain is introduced by the nonlinear geometric effect as the change of the angles between r and v struts in larger strain cannot be ignored. Upon release of the applied vertical displacement, the deformed structures recovered to their original configurations. The variation of Poisson’s ratio in large strains indicates non-linear deformations. These behaviors shows consistent tunable negative Poisson’s ratios within the same micro-architectures of the elastomeric polymer with effective stretching strain up to 20%.

**Figure 4:**
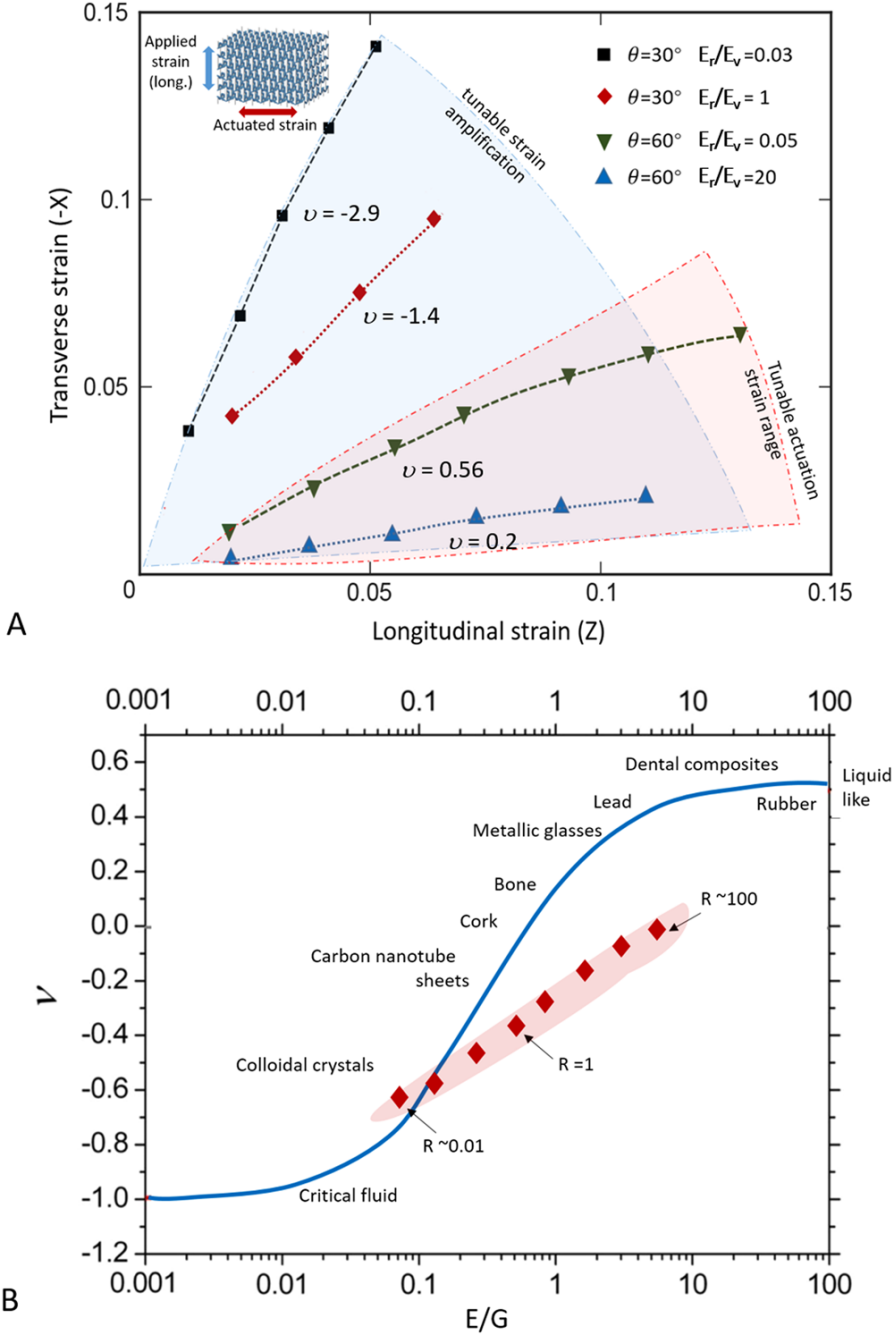
Tunable actuation strain and modulus in programmable multi-material metamaterials (**A**) Tunable transverse actuation strain tuning as a function of longitudinal strain. The negative expansion in transverse direction can be modulated through combinations of dissimilar Young’s modulus in (*r*) and (*v*) strut members within a single auxetic 3D honeycomb microlattice. (**B**) Plot of Poisson’s ratio vs. Young’s modulus/Shear modulus of architected metamaterials in comparison with bulk materials. R refers to Young’s modulus gradient within the micro-architecture of the metamaterials.

We proceeded through changing the angle between the reentrant strut and vertical strut which would change the base Poisson’s ratios when r = E_*r*_:E_*v*_ = 1. A tunable giant Poisson ratio from −7 to 0 at a uniaxial tensile strain up 20% is achieved within a single micro-architecture in MOVIE S3 and MOVIE S4. This extreme amplification of transverse strain triggered by longitudinal strain as a result from a tunablity of extreme Poisson’s ratio from *v* = 0 to −7 in a material is not possible to bulk materials or homogenous, single metamaterials. We plotted the programmable actuation in the transverse direction against the pulling strain in the longitudinal directions (Fig. 4A). It can be seen that the transverse actuation strain (Δε_x_) can be programmed through coding different moduli within microlattices, with Poisson’s ratios characterized as the slope of the Δε_x_/Δε_z_. Remarkably, through incorporation of functionally graded moduli distributions throughout the lattice, a distributed morphing deformation in the actuated transverse directions (Δx) throughout the metamaterials was observed. MOVIE S4 shows the morphing material that exhibits periodic amplified strain with Poisson’s ratio alternating from zero to negative. These metamaterials were capable of exhibiting a serpentine like actuated morphing shape as a result of the programmed stiffness throughout the lattice body. The Poisson ratio across the z direction has spatially varying values oscillating from *v = *−7 to 0, resulting in programmable strain amplifications in the orthogonal direction.

## Discussion

We have shown that a new digital light projection micro-stereolithography approach capable of assembling dissimilar materials with encoded stiffness from a few megapascal to over 600 MPa. Distributing dissimilar moduli within the microlattice can drastically change the Poisson’s ratio from zero to negative. It can be seen that despite the re-entrant angle, both negative to zero Poisson’s ratios and bulk Young’s modulus and shear modulus are tunable through encoded stiffness within the 3D architecture to a regime not achievable through homogeneous material distribution (when E_r_/E_v_ = 1). While artificial architectures have been demonstrated on single materials to achieve negative Poisson’s ratios, as shown in Fig. 4B, the programmable stiffness within architected metamaterials provide access to previously unoccupied material design space not achievable with homogenous material feedstock.

A nearly zero Poisson’s ratio can be achieved by using large ratio between base material’s Young’s modulus. These programmable modulus and morphing capabilities in 3D microlattice, in contrast to single homonegous microlattice, is owning to the disparate rigidity values distributed at vertical and loginitudal indices within the microlattice. These new material design space offered by rapid fabrication of 3D metamaterials with dissimilar material constituents open up new dimension of 3D printing of multi-materials with a large bandwidth of stiffness gradients. These auxetic materials was capable to expand transversally as large as 7 times larger than its pulling direction when axially stretched, seemingly defying the morphing capabilities observed in conventional materials. Additionally, optimization of material constituent distributions in 3D will offer new property space (negative thermal expansion, variable morphing, variable stiffness scaffold) that are not achievable by single architected material design space. Our finding suggests that adding modulus variations greatly expanded Poisson’s ratio tenability within a simple architecture, where previous techniques relying on using pure geometries to control architectures is limited to scenario where E_r_/E_v_ = 1. We envision these programmable Poisson ratio metamateriasl will find applications in directional strain amplifcations, piezoelectric metamaterials as well as designing composite lightweight metamaterials with tailored stiffness and toughness. For biological applications, these types of metamaterial with distributed modulus can be configured for guided cell growth according to different cell phenotypes with dissimilar stiffness.

## Methods

The additive manufacturing technique presented here is a high resolution, reconfigurable digital light manufacturing technique capable of printing different feedstock materials within a 3D arbitrary framework. The system incorporates a robotic resin delivery system and cleaning system to perfuse mixed resin and cleanse residue resin on substrates as well as printing interface. This allows complete cleaning of the feedstock materials without cross contaminations as indicated from the clear boundary in different colors printed within the 3D microlattice. In each layer the material switching time is 10 s including cleaning and drying time. The optical system is comprised of a digital mirror device based LED light source with a resolution of 1024 × 768 and envelope size set at 20 × 15 mm. Three linear actuators are used as the elevator for driving the platform in Z axis and motion controllers. The linear actuators contain microcontroller in the driving step motors. A micro-controller board and motor shield is used for controlling the elevator vibration motor, the fan and all other micro pumps.

The fabrication process of a single layer is illustrated in Fig. S1. Figure S2 describes the hardware setup used to realize the material change and layer stacking procedure. While pulling two clamps that holds the microlattice material along the z direction, a camera monitors the sample sidewall in the transverse direction. A customized script written in MATLAB measures the deformation of the sample in transverse and longitudinal directions.

## Electronic supplementary material

Movie S1

Movie S2

Movie S3

Movie S4

Movie S5

Supplementary material
