# Supplementary material for: Multi-material Additive Manufacturing of Metamaterials with Giant, Tailorable Negative Poisson’s Ratios
Source: Sci Rep. 2018 Jun 14;8:9139. doi: 10.1038/s41598-018-26980-7 (PMC6002359; doi:10.1038/s41598-018-26980-7)
Supplement: Supplementary file 6 — Supplementary material [file 41598_2018_26980_MOESM6_ESM.docx]

Multi-material Additive Manufacturing of Metamaterials with Giant, Tailorable Negative Poisson’s Ratios

*Da Chen, Xiaoyu (Rayne) Zheng**

*Department of Mechanical Engineering, Virginia Tech,*

*635 Prices Fork Road, Blacksburg, VA 24061, USA*

*E-mail: [raynexzheng@vt.edu](mailto:raynexzheng@vt.edu)

**S1 Robotic multi-material additive manufacturing**

A customized digital light additive manufacturing was built for producing multi-material micro-lattice described in the main text as shown in Fig. 1. The system is comprised of a Digital Micromirror Device (DMD) combined with LED UV light source and collimation assembly and imaging feedback system. Multiple projection parameters such as focal length, light intensity and distribution uniformity with in-situ image correction were employed to obtain uniform image quality on the planned focal plane. Three linear actuators are used as the elevator for driving the platform in Z-axis, motion controllers for driving the cartridge with oxygen permeable membrane in X and Y axises correspondingly. The linear actuators come with microcontroller in the integrated step motors. In the typical fabrication process, a 3D CAD model of the mulit-material metamaterials is first sliced into a series of images that represent different materials sections (Fig. S1). These 2D sliced images are transmitted to a spatial light modulator (SLM) and illuminated with UV/blue light from a light emitting diode.

To increase the use efficiency of resin material, the material transitions procedure will be conducted in every two layers rather than in every single layer. To achieve this special process, the system arranges the manufacturing sequence of various material (material A and material B, for example) as: $A_{i}-B_{i}-B_{i+1}-A_{i+1}$ rather than the intuitive $A_{i}-B_{i}-A_{i+1}-B_{i+1}$ sequence. This significantly reduced the resin switchover time, cleaning steps as well as material consumption. After the sliced image files are produced, a customized script rearranges all bmp files in a specially design sequence to be transmitted to the digital light modulator.

**S2 Calculation of effective Poisson ratios as a function of programmable stiffness**

The geometric configuration of multi-material micro-lattice is comprised of periodically repeating unit cell shown in Fig.S1 (a). The length of the re-entrant strut ($L$), the length of the vertical strut ($H$), the cross section thickness $t$(Square cross section was used here for matching experimental specimen) and the re-entrant angle ($\theta$) were considered as four primary geometrical parameters of unit cell design shown in Fig.S2(b). (Different colors represent different material constituents). Timoshenko beam theory was applied for multi-materials re-entrant structure to establish analytical solutions. Finite element analysis was also performed to verify the accuracy of analytical modeling. Bending of the re-entrant struts was considered as dominated deflection mechanism and the axial shrinkage of the re-entrant strut was ignored as well. The unit cell was assumed located in an infinite structure for the purpose of eliminating boundary effect and no torsional effect in the entire model. Considering the symmetric geometry nature of 3D re-entrant lattice, only half unit cell was needed to perform force analysis. Force decomposition of both vertical and re-entrant strut under static state is illustrated in Fig. S2 (c). The total deflection from re-entrant strut was a combination of the contribution of bending moment and shear force while the vertical strut was subjected to axial forces shown as F in Fig.S3. Note that every vertical strut on the top is shared by four adjacent unit cells, four struts on the corner should only have one-fourth load compared to central strut. Due to highly symmetry property of honeycomb structure, the 3D multi-material unit cell can be further simplified into 2D units which are marked out by red dash line. The force decomposition of 2D unit cell is shown in Fig S3(b).

From Fig. S3(b), the vertical beams only have compression force load as assumed earlier, while the re-entrant beams are subject to shear force. The values of load P and load T on re-entrant struts relative to F can be obtained from force equilibrium:

$P=\frac{F}{4}sin\theta$ (1)

$T=\frac{F}{4}\cos\theta$ (2)

Since the deformation of re-entrant strut is symmetry, obvious according to the symmetry load condition, the re-entrant strut can be further simplified to be a beam of L/2 length. This half strut can be treated as a cantilever beam which is fixed on one end and free on the other end. The fixed end is joint of re-entrant and vertical beam, and the free end is the mid-point of re-entrant beam.

Figure S3 (c) shows the half re-entrant beam in deformed state, S is the arc length direction of the beam with fixed origin point A, u is the half strut axial direction before any load applied and v is the coordinate perpendicular to original strut alignment. The $\varphi$ is the deflection angle describing the angle between original and deformed strut alignment.

The relationship between local coordinate system and the global coordinate system for re-entrant struts can be obtained from Figure S3 (c).

$\Delta x_{r} =2(\Delta u\sin\theta+\Delta v\cos\theta)$ (3)

$\Delta z_{r}=4(-\Delta u\cos\theta+\Delta v\sin\theta)$ (4)

The subscript r in equation (3) and (4) means re-entrant strut.

The compression displacement of vertical struts in z direction was written as:

$\Delta z_{v}=\frac{2FH}{E_{v} t^{2}}$ (5)

The subscript v in the equation (5) means vertical strut.

The total displacement in x and z directions for the Figure S4 (b) structure are then obtained:

$\Delta x=\Delta x_{r}$ (6)

$\Delta z=\Delta z_{r}+\Delta z_{v}$ (7)

The reentrant beam can no longer be treated as small deflection situation, large deflection cantilever beam model for displacement derivation is therefore considered. According to Figure S3(c) cantilever beam coordinate system, the moment–curvature relationship can be obtained:

$E_{r}I_{r}\frac{d\varphi}{ds}=M$ (8)

M is the moment and $\frac{d\varphi}{ds}$ is the curvature along beam arc length. The subscript r means re-entrant beam. Differentiate the moment-curvature equation with respect to s, we obtain:

$E_{r}I_{r}\frac{d^{2}\varphi}{ds^{2}}=\frac{dM}{ds}$ (9)

In the above equations, the bending moment M of given point s along arc length is:

$M\left( s \right)=P(\frac{L}{2}-\Delta u-u)$ (10)

Since $\frac{L}{2}$ and $\Delta u$ are not variables. The moment M can be differentiated by s as:

$\frac{dM}{ds}=-P\frac{du}{ds}$ (11)

Note that $cos\varphi=\frac{du}{ds}$ and take equation (11) into equation (9), we have equation (12):

$E_{r}I_{r}\frac{d^{2}\varphi}{ds^{2}}+Pcos\varphi=0$ (12)

Multiply equation (12) with $\frac{d\varphi}{ds}$, we have equation (13):

$E_{r}I_{r}\frac{d\varphi}{ds}\frac{d^{2}\varphi}{ds^{2}}+Pcos\varphi\frac{d\varphi}{ds}=0$ (13)

Further manipulate equation (13) as:

$\frac{d}{ds}\left[ \frac{1}{2}E_{r}I_{r}\left( \frac{d\varphi}{ds} \right)^{2}+Psin\varphi\right]=0$ (14)

Now integrate equation (14) from s to free end $\varphi\left( \frac{L}{2} \right)=\varphi_{\frac{L}{2}}$ , we obtain:

$\left( \frac{d\varphi}{ds} \right)^{2}=\frac{2P}{E_{r}I_{r}}(sin\varphi_{\frac{L}{2}}-sin\varphi)$ (15)

For some special design in which the re-entrant struts are not that long and slender, the shear effect should not be ignored as a part of deflection:

$\varphi=\frac{du}{ds}+\gamma$ (16)

The shear strain$\gamma$ for square cross sectional beam can be written as:

$\gamma=\frac{6P}{5G_{v}A}$ (17)

$G_{v}$ is the material shear modulus of vertical strut and A is cross sectional area of beam.

By integrating equation (15), and also notice that, when s=0 the deflection angle $\varphi\left( 0 \right)=\gamma$, we now have a function of arc length s with respect to variable $\varphi$:

$s(\varphi)=\sqrt{\frac{E_{r}I_{r}}{2P}}\int_{\gamma}^{\varphi} \frac{d\varphi}{\sqrt{sin\varphi_{\frac{L}{2}}-\sin\varphi}}$ (18)

At the free end of re-entrant beam, the arc length s of beam should be equal to L/2, and deflection angle can be assumed to be known $\varphi_{\frac{L}{2}}$:

$\frac{L}{2}=\sqrt{\frac{E_{r}I_{r}}{2P}}\int_{\gamma}^{\varphi_{\frac{L}{2}}} \frac{d\varphi}{\sqrt{sin\varphi_{\frac{L}{2}}-\sin\varphi}}$ (19)

Now define two new variables $\kappa$ and $\alpha$ as:

$\kappa=\sqrt{\frac{1+sin\varphi_{\frac{L}{2}}}{2}}$ (20)

$\sin\alpha=\sqrt{\frac{1+\sin\varphi}{1+\sin\varphi_{\frac{L}{2}}}}$ (21)

Equation (19) can be manipulated into an elliptic integral equation:

$F\left( \kappa^{2} \right)-F\left( \alpha_{0},\kappa^{2} \right): \int_{\alpha_{0}}^{\frac{\pi}{2}} \frac{1}{\sqrt{1-\kappa^{2}\sin^{2} \alpha}}d\alpha=\frac{L}{2} \sqrt{\frac{P}{E_{r}I_{r}}}$ (22)

The equation (22) is an elliptic integral function [17], and $\sin\alpha_{0}=\frac{\sqrt{1+\sin\gamma}}{\sqrt{1+\sin\varphi_{\frac{L}{2}}}}=\frac{1}{\kappa}\sqrt{\frac{1+\sin\gamma}{2}}$ . Note that $\alpha_{0}$ is also a function of $\kappa$, so the equation (22) only has one independent variable $\kappa$. The $\kappa$ value is calculated numerically in Matlab. When $\kappa$ is value is known, by calculating equation (20), we have $\varphi_{\frac{L}{2}}$.

$\cos\varphi=\frac{du}{ds}$ and $\sin\varphi=\frac{dv}{ds}$, substitute them into equation (15) then we obtain[18]:

$du=\cos\varphi ds=\sqrt{\frac{E_{r}I_{r}}{2P}}\frac{\cos\varphi}{\sqrt{\sin\varphi_{\frac{L}{2}}-\sin\varphi}}d\varphi$ (23)

$dv=\sin\varphi ds=\sqrt{\frac{E_{r}I_{r}}{2P}}\frac{\sin\varphi}{\sqrt{\sin\varphi_{\frac{L}{2}}-\sin\varphi}}d\varphi$ (24)

When $\varphi_{\frac{L}{2}}$ value is determined, integrate equation (23) and equation (24) from fix point $\gamma$ to free end $\varphi_{\frac{L}{2}}$ . The half re-entrant strut displacement $\Delta v$ and $\Delta u$ are obtained:

$\Delta u=\frac{L}{2}-\sqrt{\frac{E_{r}I_{r}}{2P}}\int_{\gamma}^{\varphi_{\frac{L}{2}}} \frac{\cos\varphi}{\sqrt{\sin\varphi_{\frac{L}{2}}-\sin\varphi}}d\varphi$ (25)

$\Delta v=\sqrt{\frac{E_{r}I_{r}}{2P}}\int_{\gamma}^{\varphi_{\frac{L}{2}}} \frac{\sin\varphi}{\sqrt{\sin\varphi_{\frac{L}{2}}-\sin\varphi}}d\varphi$ (26)

After $\Delta u$ and $\Delta v$ are obtained, the deflection of the Fig S4 (b) structure along the global coordinate $\Delta z$ and $\Delta x$ can be determined. The Poisson’s ratio of multi-material re-entrant unit cell $v_{zx}$ can be calculated as:

$\upsilon_{xz}=\text{ }-\frac{\Delta x_{r}\left( H-L\cos\theta\right)}{\left( \Delta y_{r}+\Delta y_{v} \right)L\sin\theta}$ (27)

$\upsilon_{xz}=\text{ }-\frac{\left( \frac{\text{L}^{2}}{\text{4E}_{r}t^{4}}+\frac{3}{10G_{r}t^{2}} \right)\sin\theta\left( \alpha-cos\theta\right)}{\frac{\alpha}{E_{v}t^{2}}+\left( \frac{\text{L}^{2}}{\text{4E}_{r}t^{4}}+\frac{3}{10G_{r}t^{2}} \right)\left( \sin\theta\right)^{2}}$ (28)

Where, $=H/L$ . $E_{v}$ and $\text{E}_{r}$ are the Young’s modulus of vertical and re-entrant strut respectively. $G_{r}$ is the shear modulus of re-entrant filament. Base material parameters were obtained by standard dog-bone specimen tensile test

It can be found from the equation (5) the Young’s modulus ratio ${E_{r}}/{E_{v}}$ plays an important role of describing NPR behavior of re-entrant structure. Fig. S4 plots the Poisson’s ratio as a function of the ratio between two base material stiffness and the re-entrant angle. It can be seen that despite the re-entrant angle, negative to zero Poisson ratio could be tuned to a regime not achievable through homogeneous material distribution (when ${E_{r}}/{E_{v}=1}$). A nearly zero Poisson’s ratio can be located by using large ratio of base-material’s Young’s modulus.

**S3 Finite Element Analysis**

Finite element analysis was also performed to verify our analytical model. For computational efficiency, 2-node linear beam element in space was employed in the entire model. A 10×10×5 unit cell re-entrant lattice modeled in $\mathrm{Abaqus}^{®}$ is illustrated in Fig. S5 (a). Free edge boundary condition was applied on the left and right side of entire lattice. The nodes on one end of the lattice were constrained while the nodes on top surface were subjected to an upward displacement in the y-direction. For all simulation, linear elastic material properties of both vertical and re-entrant strut were used $\theta and H/L$ ratio was ${60}^{^{\circ}}$ and 1.55 respectively. Four appropriate reference points were selected in the middle of the left, right, up and down surface to track the relative displacement to calculate the overall Poisson’s ratio. A good agreement with analytical model was graphed in Fig. 3 (main text).

**S.3 Tunable stiffness within bi-material microlattice**

The effective modulus was also investigated in this study. The new equation of overall effective modulus of multi-materials re-entrant lattice can be expressed as

$$E_{y}\text{​}=\frac{\alpha-cos\theta}{\frac{2\alpha L^{2}\left( \sin\theta\right)^{2}}{E_{v}t^{2}}+\left( \frac{L^{4}}{{2E}_{r}t^{4}}+\frac{3L^{2}}{5G_{r}t^{2}} \right)\left( \sin\theta\right)^{4}}$$

Where, $G_{r}=\frac{E_{v}}{2\left( 1+\upsilon\right)}$ and $\alpha=H/L$ (29)

Fig.S7 plots the relationship between ${E_{r}}/{E_{v}}$ (while keeping $E_{r}+E_{v}$= constant) and the largely tunable effective modulus as well as shear modulus of the lattice material within the same geometric architecture.

**Movie S1.** Bi-material microlattice with displaying negative Poisson ratio

**Movie S2.** Bi-material microlattice of the same 3D architecture displaying zero Poisson ratio

**Movie S3.** Extreme strain amplifications in metamaterial with extreme negative Poisson ratio

**Movie S4** Zero Poisson ratio metamaterial from identical 3D architecture as in Movie S3

**Movie S5.** Distributed morphing from identical 3D architecture as in Movie S3


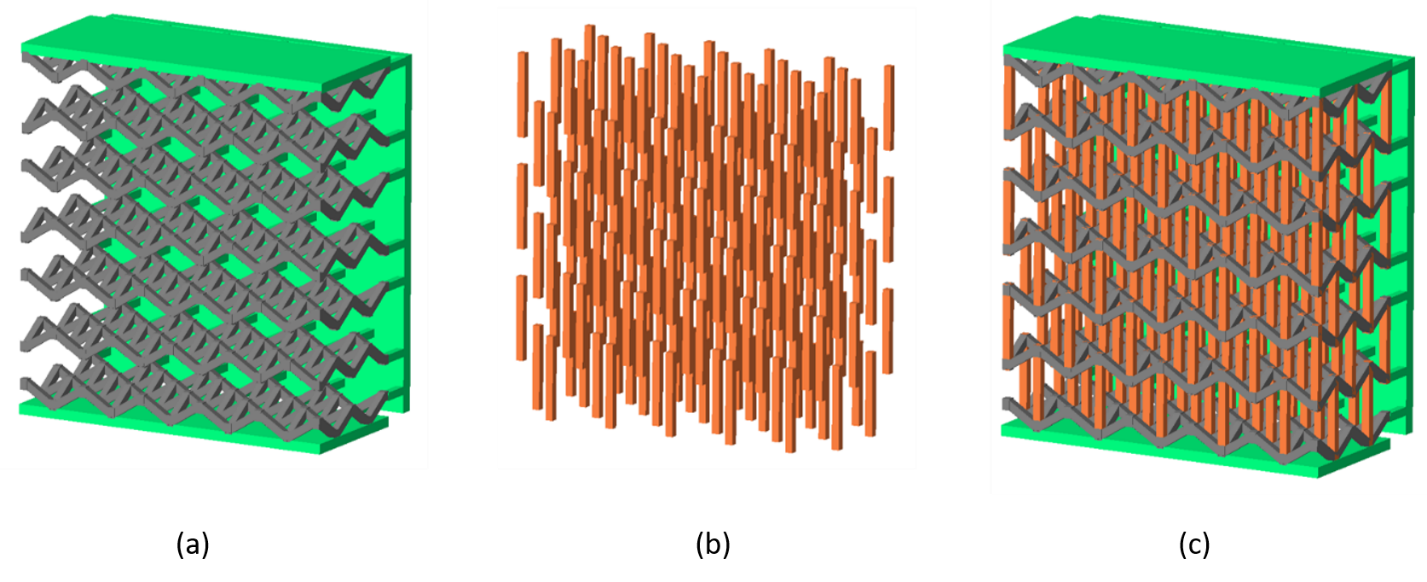


**Fig S1.** Modeling process of a multi-material lattice (a)-(b) re-entrant architecture (Material A). (b) vertical lattice section (Material B); (c) Assembled multi-material lattice;

1. (b) (c)


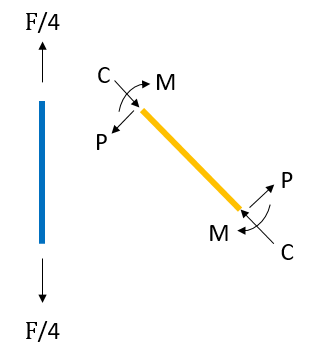

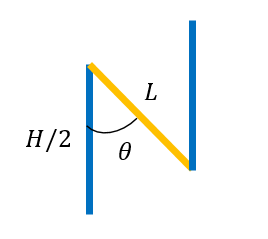

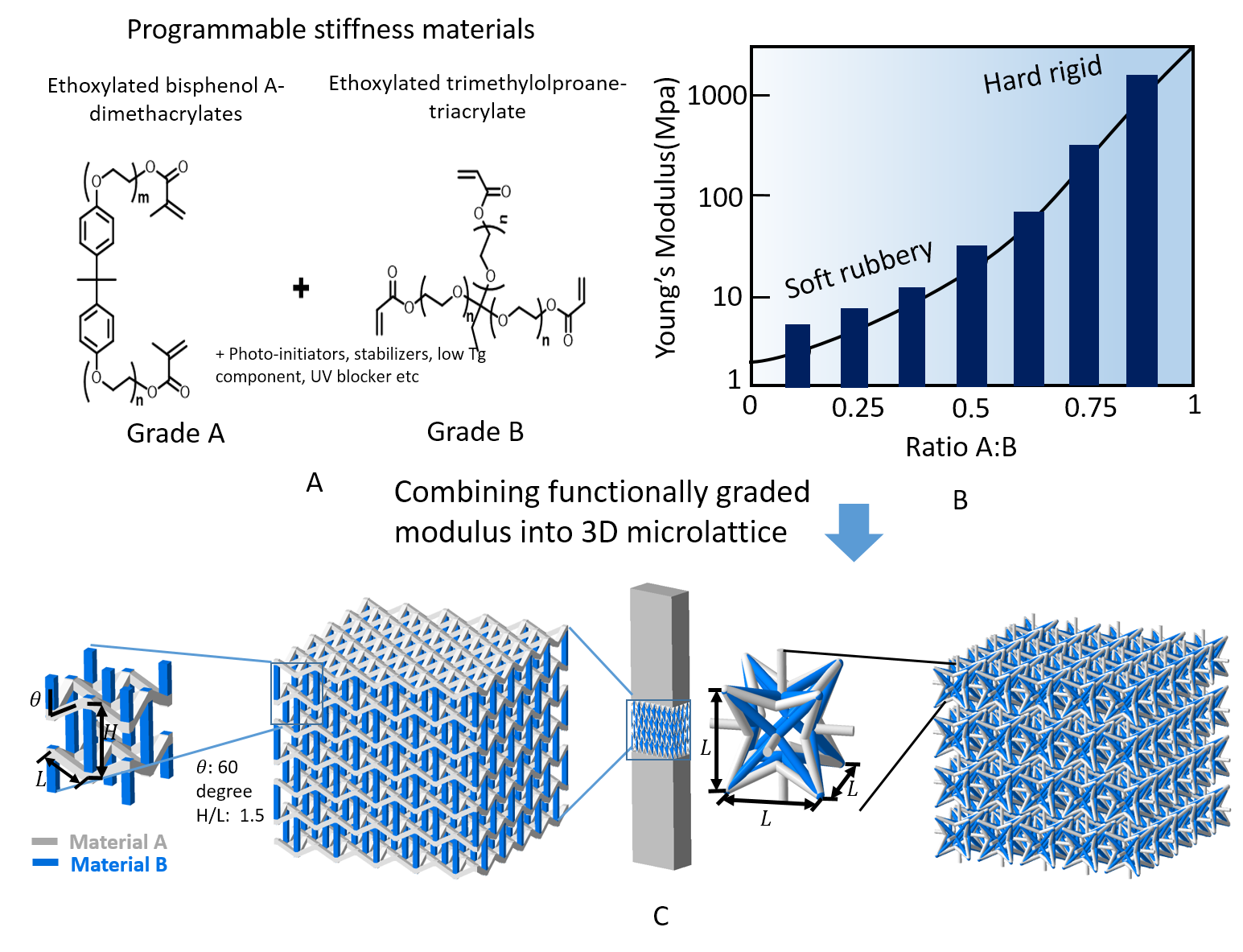


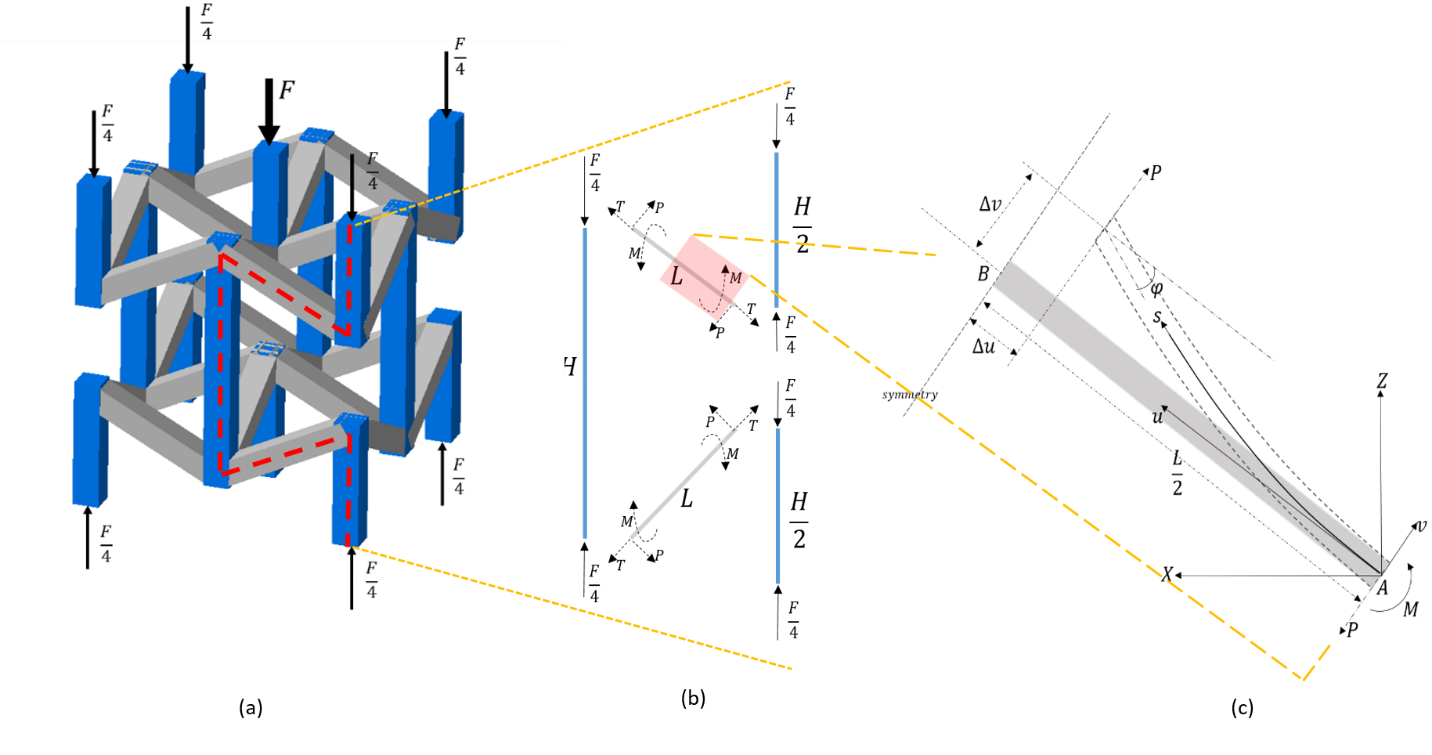
**Fig. S2 (a).** 3D re-entrant lattice structure **Fig. S2 (b).** Geometric and material parameter **Fig. S2 (c).** Force decomposition

**Fig. S3.** Deformation analysis of multi-material micro unit cell used to derive Eq. (5)

**Fig. S4.** Overall Poisson’s ratio as a function of re-entrant angle and ratio of base-material Young’s modulus $H/L=1.55$.


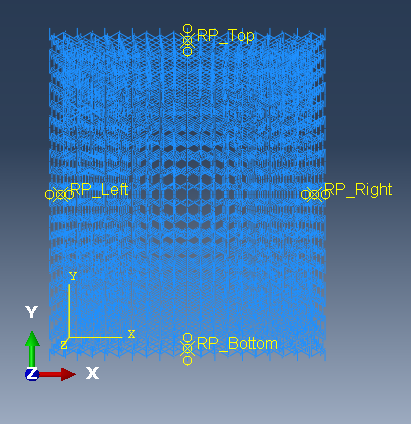

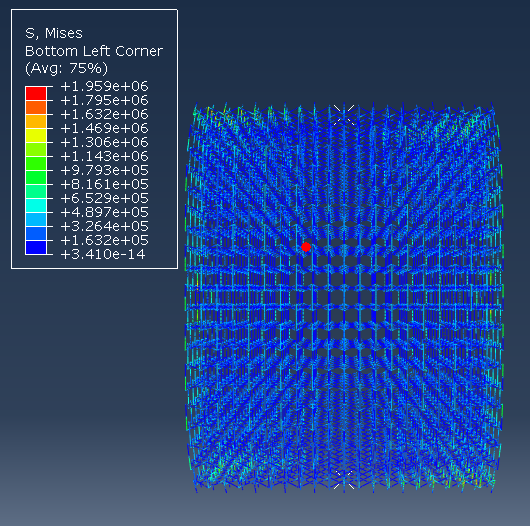


**Fig. S5 (a).** 10x10x5 3D re-entrant lattice **Fig. S5 (b).** $E_{r}/E_{v}$ = 0.011


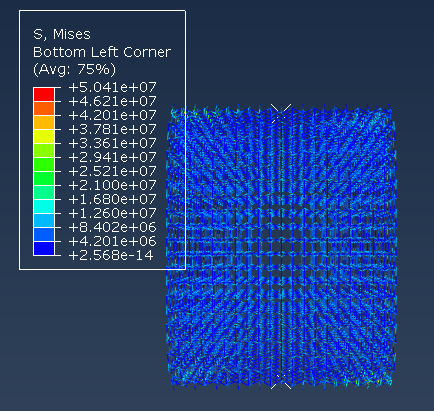

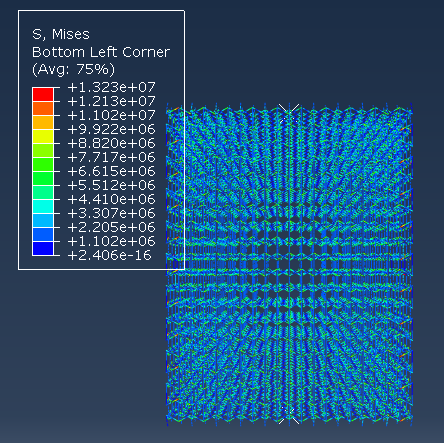


**Fig. S5 (c).** $E_{r}/E_{v}$ = 0.303 **Fig. S5 (d).** $E_{r}/E_{v}$ = 20

**Fig. S5.** Ratio of base material’s Young’s modulus effect on overall Poisson’s ratio. (a) geometric model with boundary conditions. (b)-(d) Deformation of bi-lattice material with uniaxial tensile stress with varying Young’s modulus ratios.

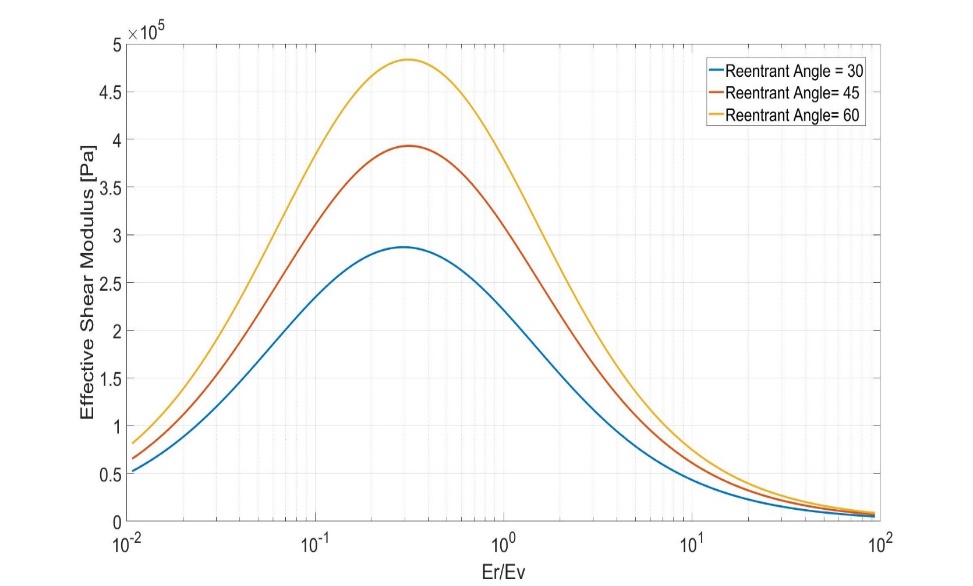


**Fig. S6.** Effective Modulus as a Young’s modulus ratio between struts members
